# Efficacy and Safety of Vaccines After Conventional Treatments for Survival of Gliomas: A Systematic Review and Meta-Analysis

**DOI:** 10.3389/or.2024.1374513

**Published:** 2024-04-19

**Authors:** Elnaz Amanzadeh Jajin, Saeed Oraee Yazdani, Alireza Zali, Abolghasem Esmaeili

**Affiliations:** ^1^ Department of Biology, Faculty of Sciences, University of Isfahan, Isfahan, Iran; ^2^ Functional Neurosurgery Research Center, Shohada Tajrish Comprehensive Neurosurgical Center of Excellence, Shahid Beheshti University of Medical Sciences, Tehran, Iran

**Keywords:** glioma, vaccine, overall survival, progression free survival, personalized vaccine

## Abstract

**Background:**

Malignant gliomas are known with poor prognosis and low rate of survival among brain tumors. Resection surgery is followed by chemotherapy and radiotherapy in treatment of gliomas which is known as the conventional treatment. However, this treatment method results in low survival rate. Vaccination has been suggested as a type of immunotherapy to increase survival rate of glioma patients. Different types of vaccines have been developed that are mainly classified in two groups including peptide vaccines and cell-based vaccines. However, there are still conflicts about which type of vaccines is more efficient for malignant glioma treatment.

**Methods:**

Phase Ⅰ/Ⅱ clinical trials which compared the efficacy and safety of various vaccines with conventional treatments were searched in databases through November 2022. Overall survival (OS) rate, progression free survival (PFS), and OS duration were used for calculation of pooled risk ratio (RR). In addition, fatigue, headache, nausea, diarrhea, and flu-like syndrome were used for evaluating the safety of vaccines therapy in glioma patients.

**Results:**

A total of twelve articles were included in the present meta-analysis. Comparison of OS rate between vaccinated groups and control groups who underwent only conventional treatments showed a significant increase in OS rate in vaccinated patients (I^2^ = 0%, RR = 11.17, 95% CI: 2.460–50.225). PFS rate was better in vaccinated glioma patients (I^2^ = 83%, RR = 2.87, 95% CI: 1.63–5.03). Assessment of safety demonstrated that skin reaction (I^2^ = 0.0%, RR = 3.654; 95% CI: 1.711–7.801, *p*-value = 0.0058) and flu-like syndrome were significantly more frequent adverse effects win vaccinated groups compared to the control group. Subgroup analysis also showed that vaccination leads to better OS duration in recurrent gliomas than primary gliomas, and in LGG than HGG (*p*-value = 0). On the other hand, personalized vaccines showed better OS duration than non-personalized vaccines (*p*-value = 0).

**Conclusion:**

Vaccination is a type of immunotherapy which shows promising efficacy in treatment of malignant glioma patients in terms of OS, PFS and duration of survival. In addition, AFTV, peptide, and dendritic cell-based vaccines are among the most efficient vaccines for gliomas. Personalized vaccines also showed considerable efficacy for glioma treatments.

## Introduction

Glioma is the most prevalent brain tumor that leads to death a few months after appearance, so that its median survival duration is 14.5 months [1]. Gliomas are classified based on the genetic profiles and variations into four different grades among which grade Ⅰ/Ⅱ are known as low grade glioma (LGG) and grades ⅠⅠⅠ/Ⅳ are known as malignant or high-grade glioma (HGG) [2]. Based on 2021 classification of gliomas, this group of tumors include CNS WHO grade 2 (Oligodendroglioma and Diffuse astrocytoma), grade 3 (Anaplastic oligodendroglioma and Anaplastic astrocytoma), and grade 4 (Glioblastoma and Astrocytoma) [3].

Management of glioma treatment develops several challenges for not only healthcare systems, but also for physicians, pharmacists, and researchers because of short survival duration and low overall survival (OS) rate [4]. Accordingly, finding effective therapeutic methods is necessary to increase OS rate and duration in glioma patients. The conventional therapeutic methods for this cancerous disease include surgery followed by temozolomide (TMZ), mTOR inhibitors, tyrosine kinase inhibitors, IDH (Isocitrate dehydrogenase) targeted therapy, and radiotherapy (RT). However, none of these methods are known as successful treatment method for increasing OS in glioma patients [5].

Accordingly, novel treatment methods are introduced for gliomas along with advancements in genomics studies and development of various targeted therapies for this disease. Vaccines are used as novel therapeutics to target specific tumor antigens and induce immune response via T cells in cancer microenvironment [6]. Several types of cancer vaccines have been introduced for cancer treatment so far, among which dendritic cell-based and peptide-based vaccines can be mentioned as the most popular ones. Dendritic cell-based vaccines are developed based on their potential of beginning primary immune response by antigen presentation, specifically in cancers [7, 8]. Peptide vaccines are produced using immunogenic tumor associated antigens (TAA) which are isolated from tumors and initiate immune response via activating T-cells leading to the lysis of cancer cells [9]. However, the main aim of developing new types of vaccines is to induce T-cell immunity and localization of this immune response to the tumor site [10]. In addition, personalized vaccines are known as promising candidates to overcome the challenges for cancer vaccination. Development of personalized vaccines have gained great attention, since it is possible to consider genetics differences of individuals in vaccine design and improve efficacy of vaccines in induction of immune response after conventional treatments and increase survival duration [11].

On the other hand, vaccines have shown promising effect on treatment of malignant gliomas because of their potential to pass through blood brain barrier (BBB) and provide targeted therapy while induce immunogenicity. Peptide-based vaccines, heat-shock protein vaccines, dendritic cell vaccines are among the most common vaccines produced for treatment of malignant glioma [6]. The use of other types of vaccines such as tumor initiating cell, B Cell Hybridoma, autologous formalin fixed vaccines, etc., has been suggested for treatment of gliomas [12–16]. On the other hand, personalized vaccines including modified genes are considered as the novel treatment which trigger genetic variations in individuals with malignant glioma [17]. These vaccines are manufactured using the genetics and epigenetics information of subjects and it is aimed to increase OS and PFS rate in glioma patients more than conventional methods of treatment and non-personalized vaccines [18].

Considering the aggressive nature of glioma tumors and the low survival rate using conventional treatment, it is important to develop novel treatment methods such as immunotherapies and vaccines to improve management of glioma patients and increase their survival. In this regard, it seems necessary to evaluate efficacy and safety of studied vaccines to compare different types of vaccines and their effects on survival od glioma patients. Based on our knowledge, no meta-analysis has been conducted for evaluation of pooled efficacy and safety of glioma vaccines and also subgroup analysis for comparison of various vaccine types. In this regard, we aim to compare efficacy and safety of studied vaccines in published articles of performed clinical trials with conventional therapeutic methods of glioblastoma. We believe that the results will provide information helpful about vaccine compartments and the outcomes of patients that can be used for designing more efficient vaccines in the future. In addition, we aim to compare the efficacy of personalized and non-personalized vaccines using survival duration in the present meta-analysis.

## Methods

### Search Strategy

We followed the strategy of Preferred reporting Initiative for Systematic review and Meta-analysis (PRISMA) guidelines in the present meta-analysis. Searching publications was done in PubMed, Cochrane Library, Embase, and ClinicalTrials.gov initiating from November 2022 and ended in March 2023. We used keywords including “glioblastoma,” “GBM,” “glioma,” “brain tumor,” “vaccine,” “dendritic cell,” “peptide vaccine,” cancer vaccine,” and “personalized” in different combinations. There was no limitation on the publication date in the screening process. In order to have a comprehensive search, the reference lists of found publications were reviewed. Two reviewers performed searching process independently and the final lists were checked while any disagreement between them was solved by a third reviewer.

### Inclusion and Exclusion Criteria

Inclusion criteria for this meta-analysis included: 1- patients diagnosed with glioma (Grade Ⅰ, Ⅱ, ⅡⅠ and Ⅳ glioma patients), 2- glioma patients treated with vaccines following surgery, chemotherapy, and radiotherapy, 3- clinical trials, 4- control or historical control groups availability, 5- control groups treatment with convention treatments, 6- overall survival (OS) rate and duration report, 7- follow-up period of at least 2 years, 8- studies published in English.

The studies which 1. were duplicates, conference letters, registered trials with no published articles, letters, or articles with no available full text, 2. were animal or cell culture studies, were excluded from this study. In case of any disagreements between researchers regarding the inclusion and exclusion criteria, it was resolved by discussing details and agreement was obtained.

### Data Extraction

Data was extracted from the finally included studies independently by two different authors. Any disagreement between authors was resolved with the presence of third author and the process continued after achieving the agreement. Author name, year, number of patients in control and vaccine groups, age, sex, overall survival rate (OS), progression free survival (PFS) rate, survival duration, tumor grade, primary or recurrent tumor, and vaccine cycles were the collected characteristics. After data collection, differences were assessed and discussed to reach agreement. For those studies which used historical control, the references were also evaluated and used for direct data extraction.

### Quality Assessment

Considering the potential errors caused by study design and implementation that can influence the quality of results in systematic review, quality of assessment is performed to reduce the risk of bias as much as possible. The risk of bias of included studies were evaluated for phase Ⅰ/Ⅱ clinical trials using Cochrane risk of bias tool, The Risk of Bias in Non-randomized Studies—Of Interventions (ROBINS-I). For quality assessment, the questions in seven domains including “Bias due to confounding,” “Bias in selection of participants into the study,” “Bias in classification of interventions,” “Bias due to deviations from intended interventions,” “Bias due to missing data,” “Bias in measurement of outcomes,” “Bias in selection of the reported results,” and overall bias. In order to achieve an exact judgment about the studies, we referred the guidelines of ROBINS-I tool [19].

### Data Analysis

Data analysis was performed using Rstudio and R (version: 4.2.1). Various packages including metaforest (version: 0.3.1), dmetar (version: 0.9000), meta (version: 6.2-0), and metafor (version: 2) were used in order to perform meta-analysis and drawing graphs. Risk Ratio (RR) was used for comparison of efficacy of vaccines to the control groups using OS rate and PFS rate as continuous data. In addition, standard mean difference (SMD) was used to compare survival duration between vaccinated and control groups. In addition, survival duration was used for subgroup meta-analyses. The significance level for effect sizes for all assessments was considered as *p* < 0.05. In addition, heterogeneity was assessed using Dixons Q-test and the *I*-squared (*I* ^*2*^) statistical tests. In case no heterogeneity was found in the results (*I* ^*2*^ < 50%, *p* ≤ 0.1), the fixed model was used for meta-analysis; whereas if the heterogeneity was high (*I* ^*2*^ < 50%, *p* ≤ 0.05), the random effect model was applied. In the following, we used subgroup analysis. The funnel plot was used for assessment of publication bias via the signs of asymmetry. Sensitivity analysis was used to evaluate the stability of the results that determines the effect of removal of each study on the meta-analysis results.

## Results

### Eligible Studies

**FIGURE 1:**
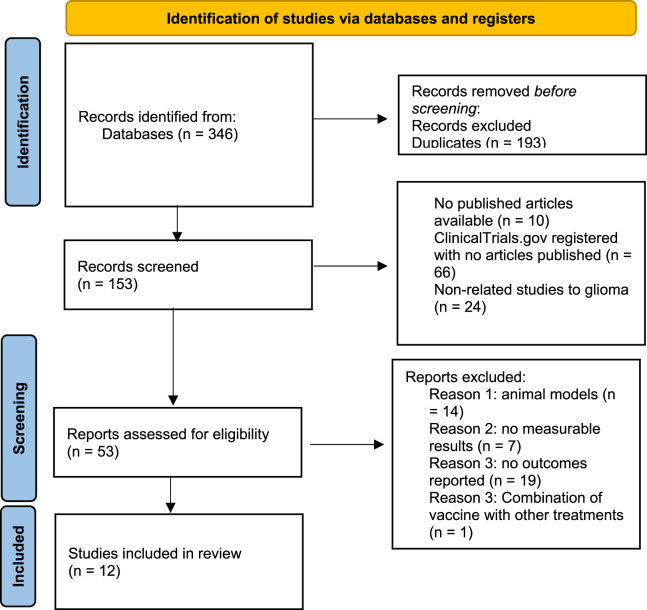
The PRISMA flow diagram.

Searching for eligible studies was done in databases (Embase, PubMed, Google Scholar, and ClinicalTrials.gov) and it yielded 346 studies, primarily. Duplicates exclusion was performed before screening leading to the removal of 193 articles. Abstract screening of remaining 153 records led to removal of 100 of them because there were no full text articles available (10 records), registered clinical trials but no articles published (66 records), and studies on vaccines of cancers other than glioma (24 records). The methodology of remaining 53 articles were reviewed and studies without measurable results and outcomes, or those which did not complete the trial were excluded. Finally, 12 phase Ⅰ/Ⅱ clinical trials met all the inclusion criteria and included in the present meta-analysis [12–16, 20–28]. The process of article selection was performed based on the PRISMA flow as shown in Figure 1.

**FIGURE 2:**
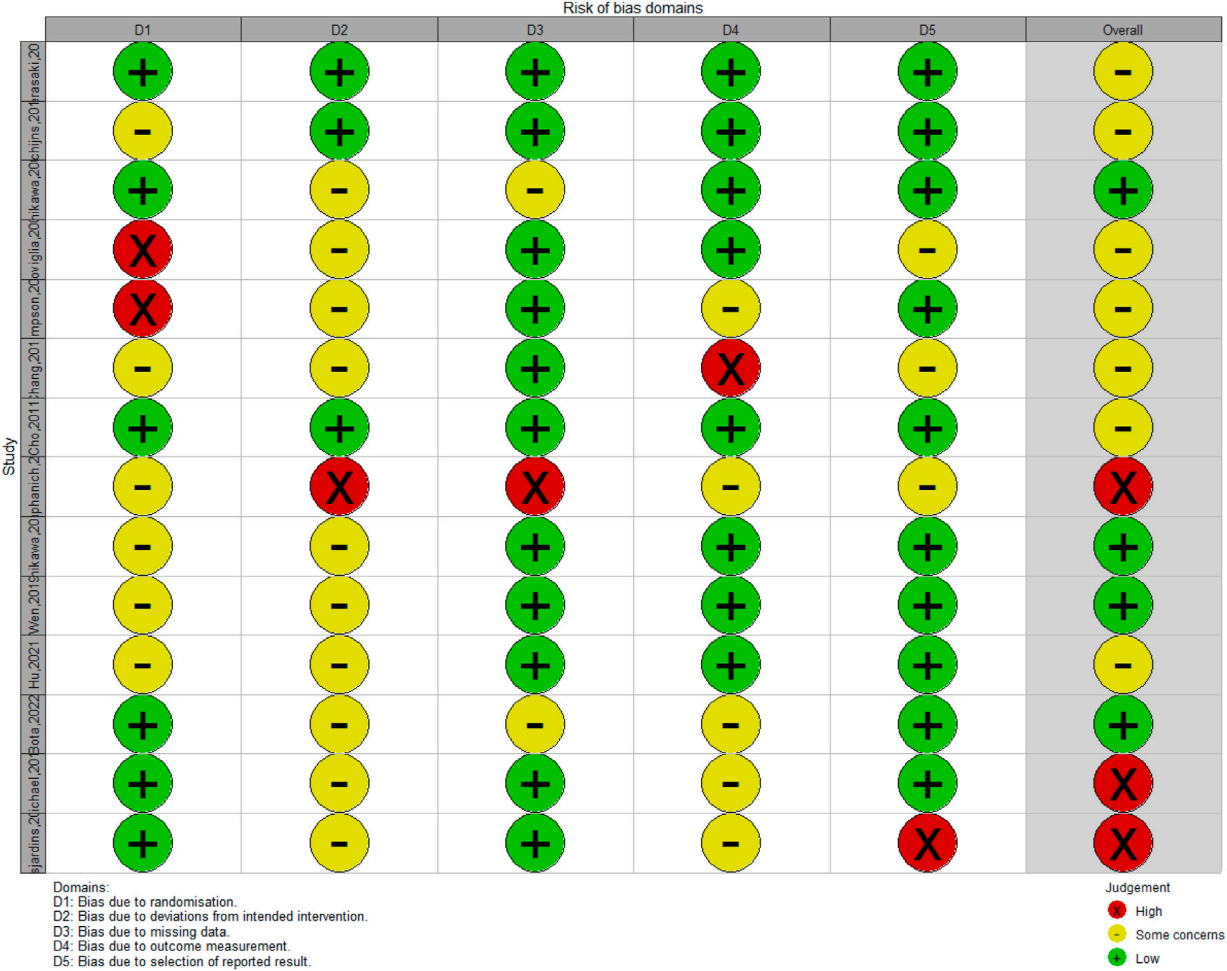
The distribution of methodology quality assessment results of included studies.

### Characteristics Collection

A total of 289 patients with glioma from different grades [68 low grade glioma (LGG), 221 high grade glioma (HGG)] were included in this study, among which 52 were recurrent and 237 were primary cases. The average Karnofsky Performance Status (KPS) of patients was 70%, while patients received normal treatments including surgery followed by radiotherapy and chemotherapy before receiving vaccines dosage. Vaccine dosage and administration showed a wide variety between included studies, so that injection cycles were variable between 1 and 20 times and similarly the duration of vaccination were variant between 1 and 24 weeks. In all included studies, vaccination was initiated at least 2 weeks after completion of routine treatments. The follow-up period after vaccination was different between 40 and 240 weeks, however, 2 years (96 weeks) follow-up was applied in most of the studies. No limitations were considered for publication date of included studies. According to the results of primary statistical analysis, no significant difference was found between age and sex of subjects between the included studies (Table 1).

**TABLE 1:** Collected characteristics from included studies.

Row	Study	Sample size	Mean age	Sex (Male %)	WHO grade	Primary or recurrent	Prior treatment	Vaccine type	Follow-up duration (Weeks)	2 years OS rate (%) Intervention/Control	Control
1	Terasaki, 2011	12	61	74.95	Ⅰ/Ⅱ	Recurrent	RT/TMZ	Peptide/PM	96	83/26	573 [42]
							RT/ACNU				
							RT/ACNU/VCR				
							Repeat Surgery, TMZ				
2	Schijns, 2015	9	50.1	55.5	ⅠⅡ/Ⅳ	Recurrent	RT	Gilovac/PM	40	77/10	39 [43]
							TMZ				
							Bevacizumab (Avastin)				
3	Ishikawa, 2007	5	50.41	33.3	ⅠⅡ/Ⅳ	Recurrent	Surgery	AFTV	96	40/0	7
							ACNU				
4	Moviglia, 2008	4	52.6	58.3	ⅠⅡ/Ⅳ	Recurrent	Surgery, RT	glioms	96	35/0	8
								ma cell B-lymphocyte hybrid (TBH)			
								mixed lymphocyte culture (MLC)			
5	Sampson, 2009	12	43.75	66.6	ⅠⅡ/Ⅳ	Primary	RT, TMZ, CW	DC	240	50/42	1578 [44]
6	Chang, 2011	16	44.5	47.05	ⅠⅡ/Ⅳ	Recurrent	Surgery	DC	138	37.5/3.2	63
7	Cho, 2011	12	52.11	44	ⅠⅡ/Ⅳ	Primary	Surgery, CCRT, Temadol	DC	132	44.4/18.75	16
8	Phuphanich, 2013	20	44.2	60	ⅠⅡ/Ⅳ	Primary/Recurrent	Surgery, RT, TMZ, Avastin	DC	160	55.6/0	—
9	Ishikawa, 2014	24	48	70.8	Ⅰ/Ⅱ	Primary	Surgery, RT, TMZ	AFTV	96	50/24	40 [45]
10	Wen, 2019	75	57.4	54.3	ⅠⅡ/Ⅳ	Primary	RT, TMZ	DC/PM	48	21/3	43
11	Hu, 2021	36	54	64.35	Ⅰ/Ⅱ	Primary/Recurrent	Surgery, RT, TMZ	DC	15	92/42 ()	369 [46]
										100/42 ()	
12	Bota, 2022	60	59	70	ⅠⅡ/Ⅳ	Primary	Surgery, TMZ, bevacizumab	TIC/PM		27/15	833 [47]
											458 [48]
											978 [49]
											573 [50]

Abbreviations: ACNU, nimustine hydrochloride; TMZ, temozolomide; RT, radiation therapy; VCR, vincristine; PM, personalized Medicine; PR, partial response; NC, no change; PD, progressive disease; DC, dendritic cell-based vaccine; NR, not reported; AFTV, autologous formalin fixed tumor vaccine; CW, carmustine wafer; CCRT, combined chemotherapy and radiotherapy; TIC, tumor initiating cell.

### Quality Assessment of Included Studies

Since all the included studies were PhaseⅠ/Ⅱ CTs, ROBINS-I tool was used for quality assessment of 13 included studies. Seven studies used historical controls as the control groups, while the remaining 5 studies had control groups. In none of the studies, randomization process was mentioned for group deviations, while all participants were aware of their treatment process. In terms of outputs, OS rate was reported in all 12 studies, but PFS rate was reported in 9 of included studies. Detailed information is mentioned in Table 1. In addition, adverse effects reported in included studies were not the same in all studies and accordingly, the adverse effects which were mentioned in more than two studies were used for safety assessment (Figure 2).

**TABLE 2:** Adverse events reported in the included studies.

Study	Flu-like symptoms (%)	Encephalopathy	Headache (%)	Skin reactions (%)	Gastrointestinal (%)	Lymphopenia (%)	Pruritus (%)
Terasaki, 2011	NR	NR	NR	50, Grade 1	NR	16.7 Grade 1	16.7, Grade 2
				33.3 Grade 2			
				16.7, Grade 3			
Schijns, 2015	NR	NR	22	60, Grade 1	NR	NR	NR
Ishikawa, 2007	66.7, Grade 2	NR	NR	22.2, Grade 2	NR	NR	NR
Moviglia, 2008	50, Grade 1	16.7, Grade 1	NR	NR	NR	NR	NR
		33.4, Grade 2					
		16.7, Grade 4					
Sampson, 2009	NR	NR	NR	33.3, Grade 1	NR	NR	NR
Chang, 2011	NR	NR	NR	NR	NR	29.5, Grade 1 and 2	NR
						29.5, Grade 3 and 4	
Cho, 2011	NR	NR	NR	NR	NR	5.9, Grade 1	NR
Phuphanich, 2013	36, Grade 1	NR	NR	9, Grade 1	18, Grade 2	NR	18, Grade 2
Ishikawa, 2014	2, Grade 1	NR	NR	21, Grade 1 and 2	7, Grade 1 and 2	8, Grade 1 and 2	2.8, Grade 1
Wen, 2019	NR	57.5, Grade 2 and 3	8.8, Grade 1	NR	21.3, Grade 1	NR	NR
Hu, 2021	16.7, Grade 1 and 2	NR	NR	50, Grade 1 and 3	2.8, Grade 1	NR	NR
Bota, 2022	54.4, Grade 1 and 2	36.8, Grade 1 and 2	NR	15.8, Grade 1 and 2	38, Grade 1 and 2	NR	10.5, Grade 1

### Efficacy

#### OS

**FIGURE 3:**
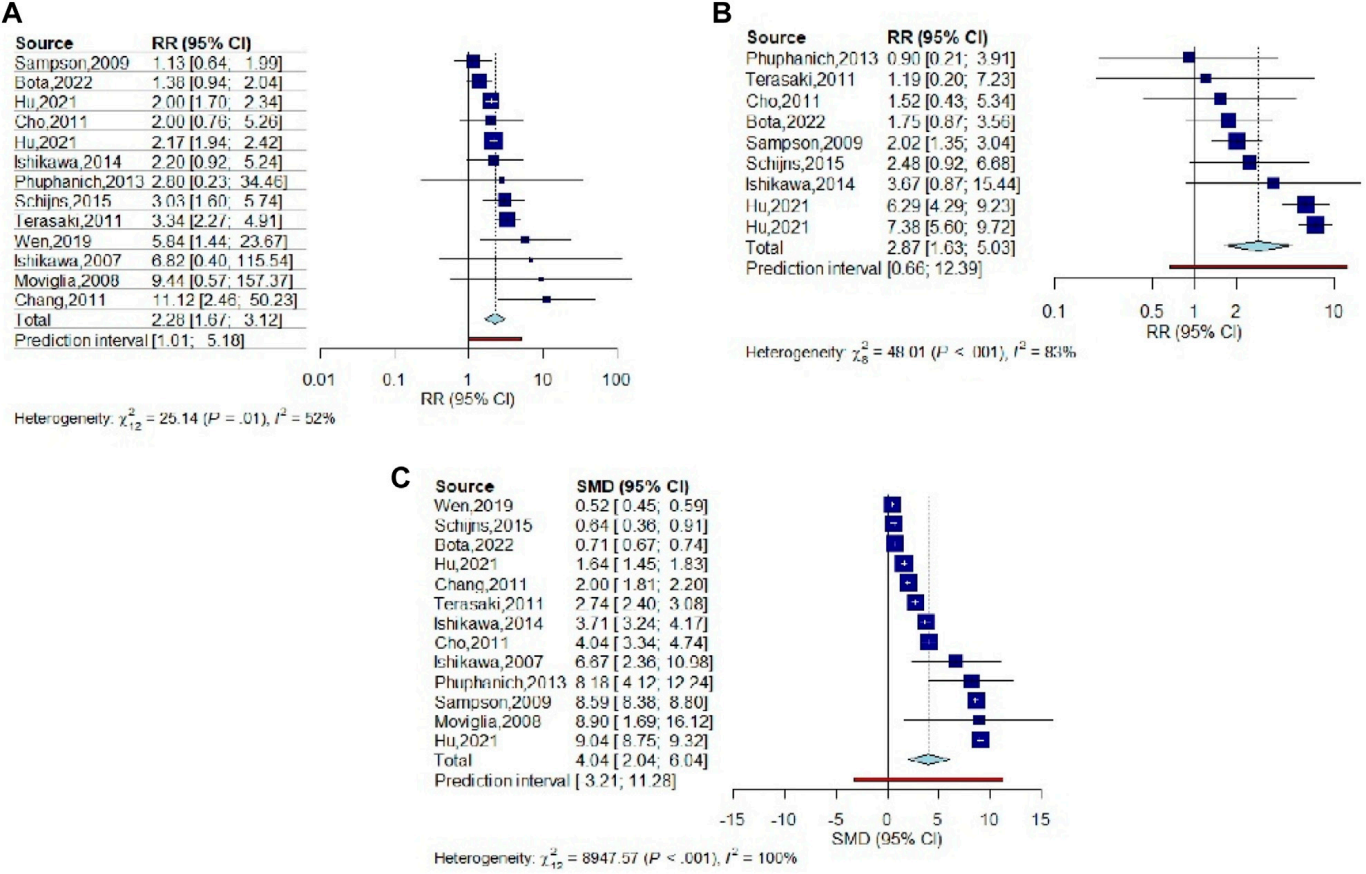
Forest plot for RR of OS rate in vaccinated glioma patients. **(A)** Meta-analysis of OS rate for 2 years follow-up (RR: 2.283, 95% CI: 1.671–3.118), **(B)** Meta-analysis of PFS rate for 2 years follow-up (RR: 2.866, 95% CI: 1.634–5.028), and **(C)** Meta-analysis of survival duration for vaccinated GBM patients compared to unvaccinated patients (SMD: 6.845, 95% CI: 2.676–11.014).

The rate of OS was used to evaluate the efficacy of vaccination in the included studies within 2 years follow-up. Accordingly, OS rate for 2 years follow-up was extracted for the studies which reported even longer follow-up time. Results of the meta-analysis revealed a median heterogeneity rate (*I* ^*2*^ = 52.3%), while the risk ratio (RR) was obtained equal to 2.283 (95% CI: 1.671–3.118) indicating that vaccination leads to increase in the number of survived patients within 2 years follow-up significantly (*p*-value < 0.0001) (Figure 3A). Assessment of RR for each of included studies revealed that highest RR was found for [22] (RR = 11.17, 95% CI: 2.460–50.225) who reported 37.5% of OS rate after 2-year follow-up and 18% OS rate after 5-year follow-up, and lowest RR was obtained for [16] (RR = 1.380, 95% CI: 0.936–2.035) who reported 27% OSR after 2 years follow-up.

#### PFS

In addition, progression free survival (PFS) rate was used to evaluate the efficacy of vaccination in recurrence of glioma. According to the results, pooled RR of PFS rate for nine studies was obtained as 2.866 (95% CI: 1.634–5.028), meaning that PFS rate in patients treated with vaccines after conventional treatment within 2 years follow up was significantly higher than patients who were treated only with conventional treatments (*p*-value < 0.001), though heterogeneity for this analysis was high (*I* ^*2*^ = 83.3%) (Figure 3B).

#### Survival Duration

Survival duration was also used for meta-analysis and evaluate the efficacy of studied vaccines. Standard mean difference (SMD) was used to compare survival duration between vaccinated and control or historical control groups for each study and pooled SMD was obtained as 6.845 (95% CI: 2.676–11.014). This result shows a significant positive effect of vaccination on survival time in comparison with patients who underwent only conventional treatment (*p*-value < 0.001) (Figure 3C). According to the results, the biggest SMD was reported by [12] as 8.904, [21] as 8.594, and [23] as 8.179, who reported the highest survival duration as 25, 28.19, and 38.4 months, respectively.

#### Safety

Safety assessment was performed in this meta-analysis using the rate of Grade 1–3 of Treatment Related Adverse Events (TRAEs) including fever, headache, flu-like symptoms, lymphopenia, injection site reactions, vomiting, and diarrhea. The extracted data for safety assessment has been summarized in Table 2.

**FIGURE 4:**
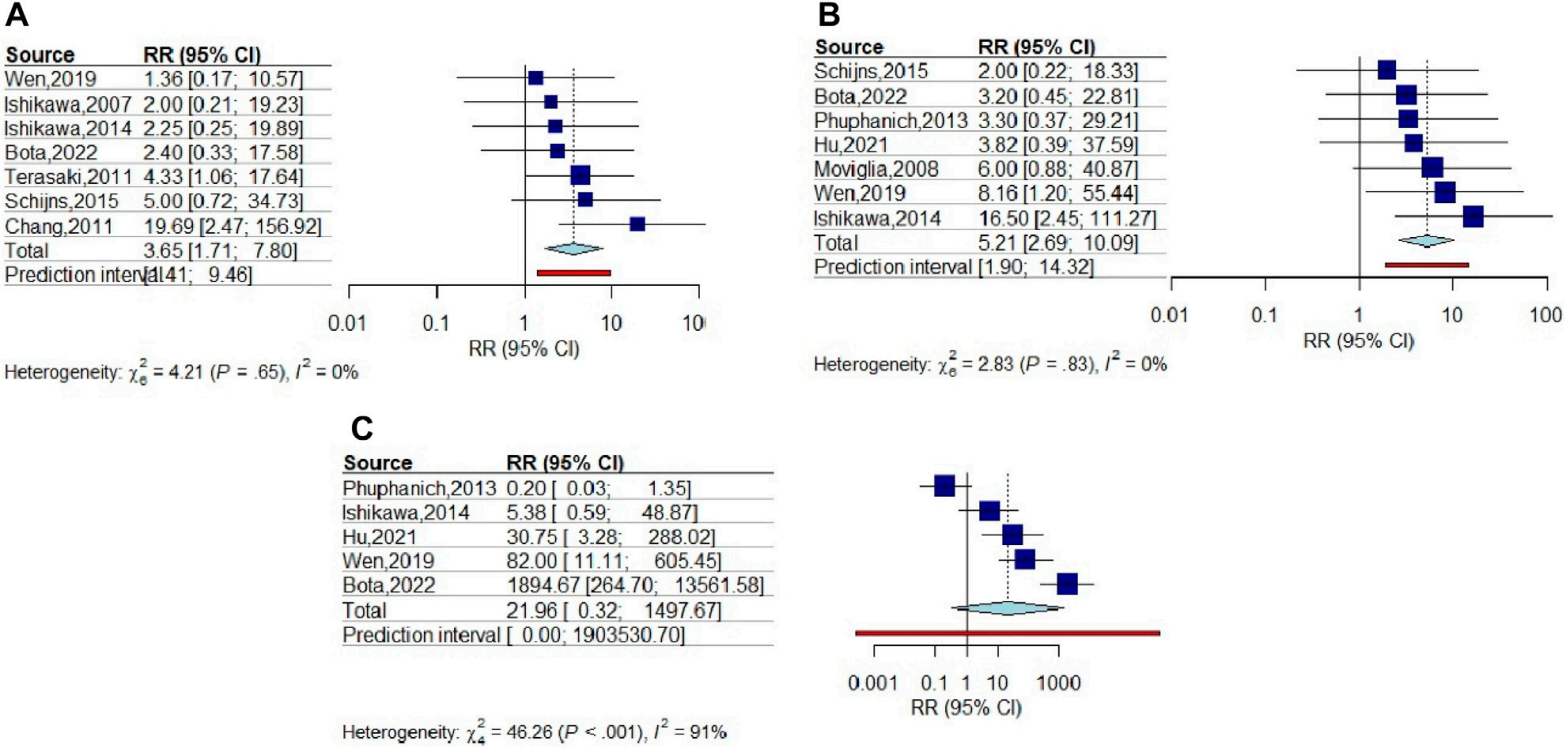
Forest plots of skin reaction, flu-like syndrome, and gastrointestinal symptoms after vaccination of glioma patients. **(A)** Meta-analysis of skin reaction (RR = 3.654; 95% CI: 1.711–7.801), **(B)** Meta-analysis of flu-like syndrome (RR = 5.21, 95% CI: 2.691–10.086), and **(C)** Meta-analysis of lymphopenia (RR = 21.962, 95% CI: 0.322–1497.67). For all the analyses, the significance level of *p* ≤ 0.05 was considered.

Meta-analysis of skin reaction after vaccination showed low between-study heterogeneity (*I* ^*2*^ = 0.0%). The pooled RR for skin reaction was higher than control groups (RR = 3.654; 95% CI: 1.711–7.801), and it was statistically significant (*p*-value = 0.0058) (Figure 4A). Assessment of pooled RR for flu-like syndrome revealed low between-study heterogeneity (*I* ^*2*^ = 0.0%), while this adverse effect was significantly higher in vaccinated groups compared to the control groups (RR = 5.21, 95% CI: 2.691–10.086) (*p*-value = 0.0009) (Figure 4B). RR was also calculated for lymphopenia which was considered as a serious problem after vaccination and the results revealed high between-study heterogeneity (*I* ^*2*^ = 91.4%). The obtained results demonstrated that gastrointestinal symptoms do not appear significantly more frequent than the control group (RR = 21.962, 95% CI: 0.322–1497.67) (*p*-value = 0.112) (Figure 4C).

### Subgroup Analysis

Sensitivity analysis results did not demonstrate significant differences omitting each of studies. Accordingly, subgroup analysis was performed based on all the factors that have the potential to influence the results of efficiency of the vaccines. Subgroup analysis was performed based on the different factors including vaccine type, glioma grade, primary or recurrent tumor, and personalized vaccine characteristics.

#### Vaccine Type

**FIGURE 5:**
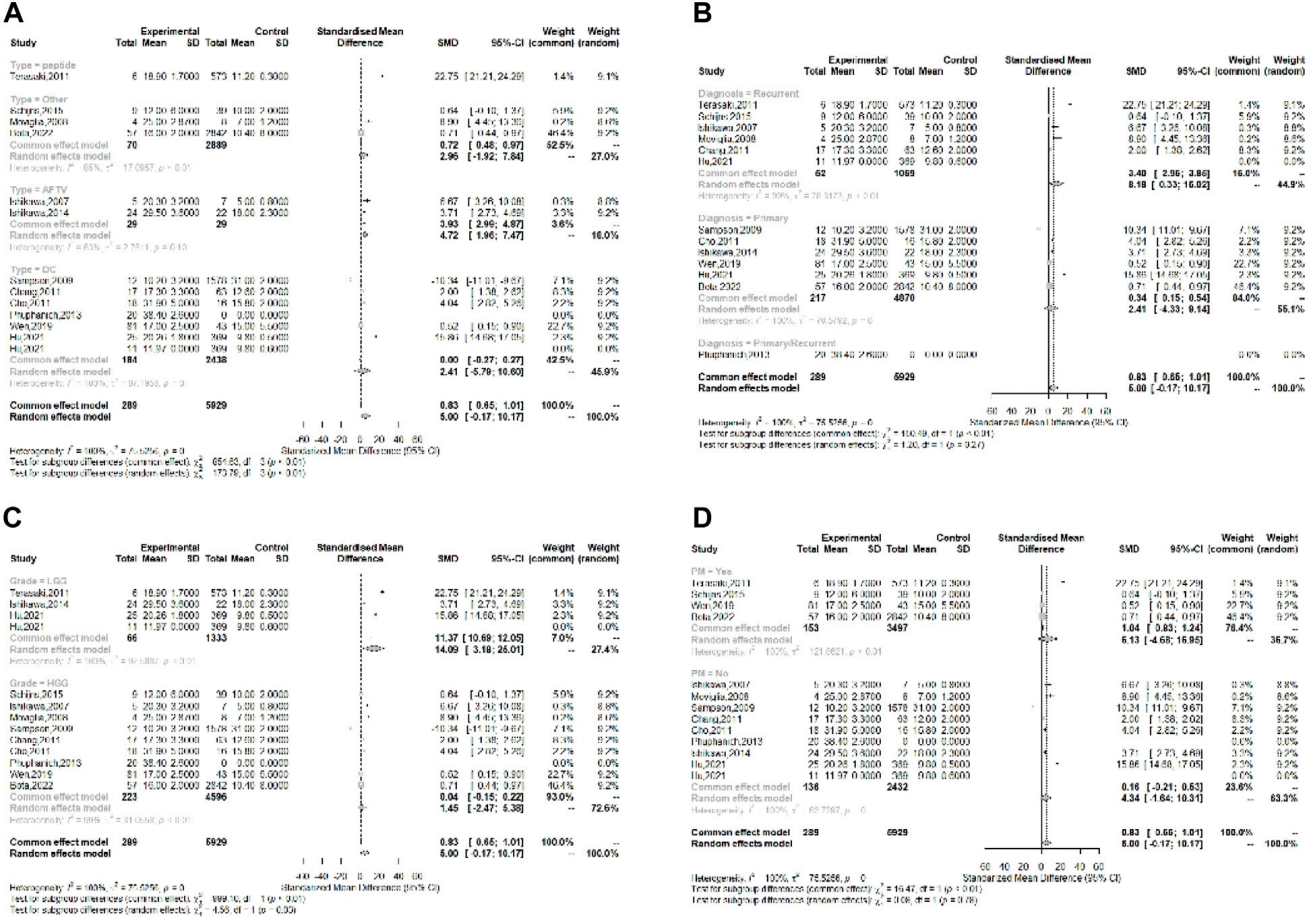
Subgroup Forest plot of OS duration based on the subgroups including **(A)** Vaccine types (peptide (SMD = 22.75), AFTV (SMD = 4.72), DC (SMD = 2.41), and others (SMD = 2.69), **(B)** Tumor diagnosis (Primary (SMD = 8.18) and Recurrent (SMD = 2.41)), **(C)** Tumor Grade (HGG (SMD = 14.09) and LGG (SMD = 1.45)), and **(D)** Personalized approach for vaccine production (Personalized (SMD = 6.13), and Non-Personalized (SMD = 4.34)).

In the subgroup of vaccine type, mean survival duration was compared between vaccinated and control groups in four subgroups including peptide, AFTV, DC, and other (including Gliovac, B cell hydroma, and tumor initiating cell). The pooled SMD for peptide, DC, and other subgroups revealed that vaccination increased survival duration in all subgroups (22.75, 2.96, and 2.41, respectively) (*p*-value < 0.01); however, although pooled SMD for AFTV showed increase in mean survival time, the difference was not statistically significant (SMD = 4.72, *p*-value = 0.1) (Figure 5A).

#### Tumor Type

The subgroup analysis was performed to assess the efficacy of vaccination on primary and recurrent tumors, separately. According to the results, pooled SMD for recurrent subgroup was highly effective (SMD = 8.148) and this effect was statistically significant (*p*-value < 0.01). The results of this subgroup analysis for primary tumors showed smaller SMD (SMD = 2.41) than recurrent subgroup, but it was still significant (*p*-value = 0), indicating that vaccination is also effective for primary tumors (Figure 5B). These results mean that vaccination leads to drastically longer survival duration in recurrent glioma patients compared to those who undergone only conventional treatments.

#### Glioma Grade

Assessment of vaccination efficacy based on the tumor grade was also performed. For this analysis, WHO grade Ⅰ/Ⅱ glioma were classified in low grade glioma (LGG) group and WHO grade ⅡⅠ/Ⅳ were classified in the high-grade glioma (HGG) group. The results of SMD for survival duration showed that in HGG subgroup, vaccination has led to increase in survival duration (SMD = 14.09, *p*-value < 0.01) more effectively than LGG subgroup (SMD = 1.45, *p*-value < 0.01). Noteworthy, the pooled SMD for both groups are statistically significant (Figure 5C).

#### Personalized Approach

The efficacy assessment of vaccines which were manufactured based on personalized approaches was performed in PM subgroup analysis. The results of this subgroup analysis demonstrated that PM vaccines lead to longer survival duration than control groups who underwent only conventional treatments (SMD = 6.13, *p*-value < 0.01); however, the pooled SMD for non-PM vaccines was slightly smaller than PM vaccines but it also demonstrated statistically significant effect on survival duration (SMD = 4.34, *p*-value < 0.01) (Figure 5D).

### Publication Bias

**FIGURE 6:**
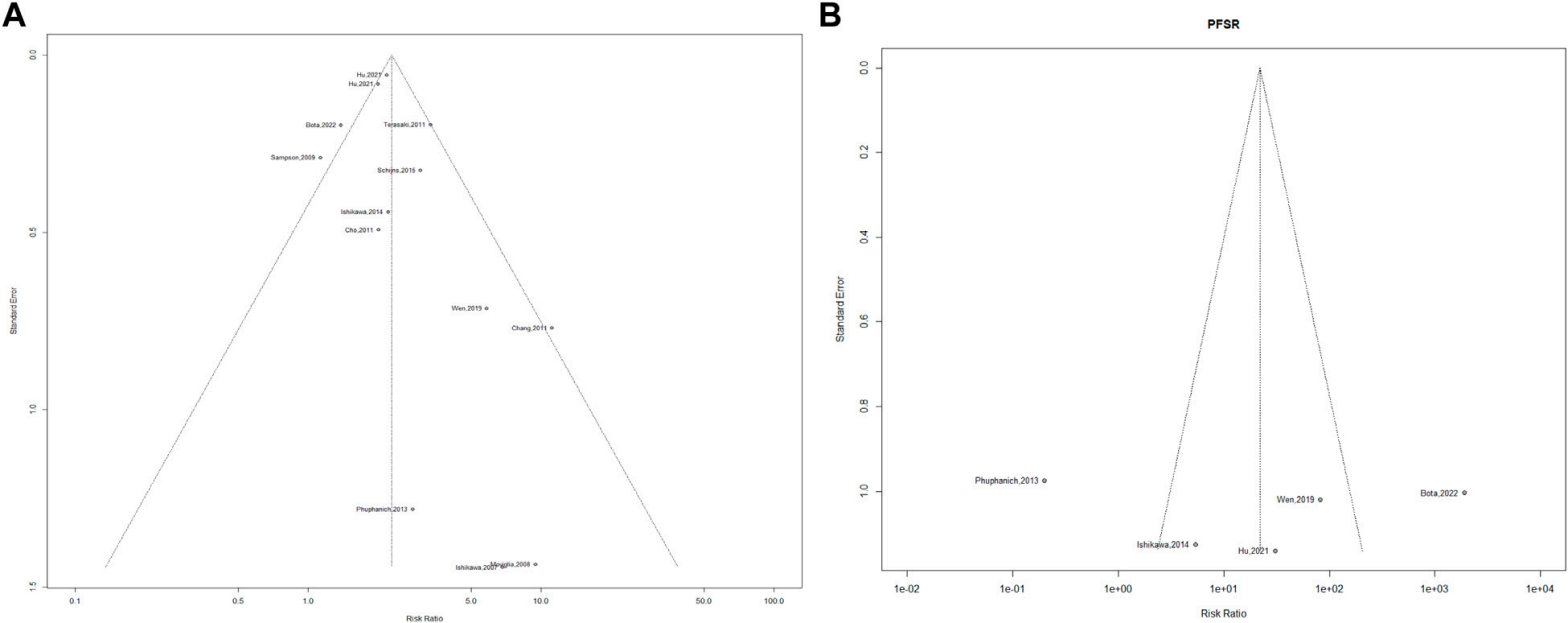
Publication bias. **(A)** OS, Beggs test, *p*-value = 0.032, **(B)** PFS, Beggs test, *p*-value = 0.390.

Funnel plot analysis for assessment of vaccine effectiveness on the OS rate and PFS rate showed a significant asymmetric distribution (Figures 6A, B). The Egger linear regression test results revealed no potential publication bias for OS rate (I^2^ = 49.84%, H^2^ = 1.99, *R* ^2^ = 57.87%) and PFS rate (I^2^ = 67.11%, H^2^ = 3.04, *R* ^2^ = 0.00%) between included studies.

## Discussion

Based on our knowledge, the present meta-analysis is the first one which evaluates the effectiveness of various types of vaccines against gliomas. Although it has been mentioned that immunotherapy using vaccines is an effective method for gliomas, there are still uncertainties about their efficacy and advantages above conventional treatments [17]. Previous reviews have discussed the efficacy of glioma vaccines in terms of immune responses and cytotoxicity and the need for longer terms of assessments and larger populations are emphasized [29]. In general, the pooled RR in this study showed that not only 2-year OS rate, but also PFS rate in vaccinated groups was significantly higher than conventional treatments. In addition, we showed that OS rate of vaccinated patients, without considering the type of vaccine, was higher than patients treated using conventional methods.

Peptide vaccines are known as effective candidates for glioma immunotherapy, because they can be used for specific targets in cells such as mutated and overexpressed genes and result in improved prognosis in malignant glioma [30, 31]. The previous studies showed that targeting key oncogenes in malignant gliomas as an effective immunotherapy method for longer survival duration and higher OS rate while mild adverse effects are observed after vaccination [32]. Our results of subgroup analysis showed that peptide vaccines result in a significantly longer survival duration in comparison with patients treated only with usual methods. Noteworthy, designing peptide vaccines using novel methods such as machine learning and deep learning algorithms along with specific delivery systems for these vaccines in order to increase the immunogenicity still remain as challenging concerns about peptide vaccines [33]. Considering the potential of peptide vaccines, designing personalized peptide vaccines via genomics data of patients seems a necessity in cancer vaccines which should be focused on more [34].

Dendritic cell vaccines are known as promising vaccines for induction of immune response in glioma cells and also increase their sensitivity for chemotherapy [35]. In this regard, Wang et al. meta-analyzed six clinical trials to evaluate the efficacy of dendritic cell-based vaccines for malignant glioma and their results showed longer survival duration and 2 years survival rate for vaccinated patients [36]. The efficacy of antigen pulsed dendritic cell was meta-analyzed for HGG patients and their results showed no improvement in neither KPS performance nor lymphocytes percentages, while INFɣ showed higher level in DC treated patients [37]. The results of this study challenged the results of previous meta-analysis and led to rise more uncertainties in terms of DC-based vaccines. However, a current meta-analysis showed efficacy of DC therapy for malignant gliomas and immune responses via induction of CD8^+^ T cells, while they also reported fatigue as most common adverse effects of this treatment method [38]. Considering that these studies focused on only DC-based therapies, these vaccines were not compared to other vaccines before. Our results of subgroup analysis showed that although DC-based vaccines are among the most common vaccines against malignant glioma, but the increase in survival duration in DC-based vaccine group was smaller than other vaccine types. Accordingly, it seems that more clinical trials with larger populations are required to compare the effects of these vaccines with other types of vaccines.

The effectiveness of autologous formalin fixed vaccines has been shown for various cancers such as hepatocellular carcinoma, breast cancer, etc. The results of these studies showed promising efficacy for AFTV for treatments of cancers and especially metastatic cancers which lead to higher OS and PFS rates. In the present meta-analysis, AFTV showed large increase in OS and PFS rates and also leads to longer survival duration. Comparison of AFTV with other vaccines showed better effect on survival duration than CD-based vaccines and other vaccines which included B cell Hydroma and TIC vaccines. Altogether, these findings shed light on the more efficient compartments of the vaccines on the survival of glioma patients. On the other hand, the results obtained in all types of studies showed better OS rate than conventional treatments. These findings show the potential of vaccines to be added to the conventional treatment process of glioma patients in order to increase survival duration of patients. However, more studies and phase Ⅱ and pahseⅡⅠ clinical trials are required to show the reliability of vaccines in terms of efficacy and safety using bogger populations.

In terms of safety, meta-analysis of adverse effects including flu-like syndrome, gastrointestinal symptoms, and skin reaction for vaccination in comparison with patients who underwent only usual treatments showed that the frequency of skin reaction and flu-like syndrome was not significantly more in vaccinated patients than control groups. However, gastrointestinal symptoms showed higher prevalence in vaccinated patients in comparison with control groups. Considering that vomiting and diarrhea can lead to serious problems in glioma patients and disturb vaccination process, improvement of vaccines formulation in order to reduce gastrointestinal symptoms seems necessary for future vaccines.

Personalized medicine, as the novel and growing area in medicine, is known as a hope for development of efficient treatment methods for cancer treatment. Personalized vaccines are designed based on the genomics data of each patient and show efficacy and safety for patients [39]. Despite the glory of personalized medicine, the application of this immunotherapy method has several challenges such as optimized dosage, potential adverse effects, factors influencing immune response, etc [40]. Accordingly, lots of uncertainties arise related to personalized vaccines. In the present study, we performed a subgroup analysis to compare the effect of conventional vaccines and personalized vaccines on the survival duration in glioma patients. The results showed that although the number of studies which used personalized vaccines and accordingly, the number of subjects were less than conventional vaccines, but pooled SMD for personalized vaccines was higher than conventional vaccines. Noteworthy, these results obtained for a heterogenous vaccines group including DC-based, AFTV, and peptide vaccines. Therefore, we claim that personalized vaccines show promising effects on survival duration in glioma patients; however, we propose studying the effect of personalized vaccines on larger populations in randomized clinical trials to compare various types of personalized vaccines in terms of efficacy and safety.

We faced limitations in terms of number of subjects enrolled in the included studies, comparison of present results with historical controls. Considering that included studies were mainly in early phase clinical trials, this limitation caused a challenge in terms of study population. In addition, not all the included studies reported adverse effects. It is suggested to add all types of adverse effects into the studies in the future and report all versions of adverse effects. In addition, it is important to consider the severity and frequency of the adverse effects of vaccines in the future studies, since almost all mentioned studies reported the number of patients who showed or did not show adverse effects. It has been mentioned that using vaccines against glioma has shown efficacy; however, conducting phase ⅡⅠ clinical trials which bring certainty into the field of vaccine therapy for glioma is necessary [34, 41]. In addition, since vaccination is a type of immunotherapy, lymphocyte percentage data and other related assessments of immunogenicity of vaccines were rarely considered in the assessed studies, while it seems highly important for comparison of vaccines and their efficacy. It remains as another question which should be addressed in the future clinical trials. We suggest more studies in the future based on the findings of the present meta-analysis to compare different vaccine types or compartments of vaccines on the survival duration of glioma patients. We believe that our findings are highly valuable for future studies, but more comprehensive studies with larger populations are required to prove the results of the present systematic review and meta-analysis.

## Conclusion

In conclusion, the present meta-analysis explains the efficacy and safety of available vaccines and personalized vaccines for treatment of malignant glioma patients in comparison with patients who underwent only conventional treatments including RT, chemotherapy, or TMZ after surgery. We also showed higher efficacy of personalized vaccines than conventional vaccines in increasing OS in glioma patients. Peptide vaccines and dendritic cell-based vaccines are among the most popular vaccines for malignant glioma, but novel vaccines are also being developed with high efficacy. Based on the results of this meta-analysis, designing personalized vaccines and evaluation of efficacy for vaccine treatments in Phase ⅠⅡ clinical trials are suggested.

## Data Availability

Data presented in this article was extracted from articles and they were used for meta-analysis in this systematic review and meta-analysis. Accordingly there is no repository or link for this data. The articles used in this study are listed in the article.
